# Enhanced chromium (VI) removal using activated carbon modified by zero valent iron and silver bimetallic nanoparticles

**DOI:** 10.1186/s40201-014-0115-5

**Published:** 2014-08-21

**Authors:** Babak Kakavandi, Roshanak Rezaei Kalantary, Mahdi Farzadkia, Amir Hossein Mahvi, Ali Esrafili, Ali Azari, Ahmad Reza Yari, Allah Bakhsh Javid

**Affiliations:** Department of Environmental Health Engineering, School of Public Health, Ahvaz, Jundishapur University of Medical Sciences, Ahvaz, Iran; Department of Environmental Health Engineering, School of Public Health, Iran University of Medical Sciences, Tehran, Iran; Department of Environmental Health Engineering, School of Public Health, Tehran University of Medical Sciences, Tehran, Iran; Center for Solid Waste Research, Institute for Environmental Research, Tehran University of Medical Sciences, Tehran, Iran; Department of Environmental Health Engineering, School of Public Health, Qom University of Medical Sciences, Qom, Iran; Department of Environmental Health Engineering, School of Public Health, Shahrood University of Medical Sciences, Semnan, Iran

**Keywords:** Bimetallic, Chromium, Activated carbon, nZVI, Adsorption

## Abstract

Recently, adsorption process has been introduced as a favorable and effective technique for the removal of metal ions from aqueous solutions. In the present study, bimetallic nanoparticles consisting of zero valent iron and silver were loaded on the activated carbon powder for the preparation of a new adsorbent (PAC-Fe^o^/Ag). The above adsorbent was characterized by using XRD, SEM and TEM techniqes. Experimental data were exploited for kinetic, equilibrium and thermodynamic evaluations related to the adsorption processes. The Cr(VI) adsorption process was found to be favorable at pH 3 and it reached equilibrium state within 60 min. The stirring rate did not have a significant effect on the adsorption efficiency. Furthermore, the monolayer adsorption capacity of Cr(VI) based on the Langmuir model was measured to be 100 mg/g. The experimental equilibrium data were fitted to the Freundlich adsorption and pseudo second-order models. According to the thermodynamic study, the adsorption process was spontaneous and endothermic in nature, indicating the adsorption capacity increases with increasing the temperature. The results also revealed that the synthesized composite can be potentially applied as a magnetic adsorbent to remove Cr(VI) contaminants from aqueous solutions.

## Introduction

Along with industries’ development, the contaminants originating from their activities have significantly increased. Heavy metals are the main pollutants end up to the environment by these activities. High toxicity of these metals causes serious problems to the ecosystem even at low concentrations [1]. Chromium (Cr) is one of the most dangerous heavy metals that has many applications in the metal cleaning and plating baths, painting, tannery and fertilizer industries [2]. Cr mainly exists in two stable oxidation states, Cr(VI) and Cr (III). Cr(VI) form is more toxic to living things than Cr(III) due to its carcinogenicity, toxicity and high aqueous solubility [1,3]. When the concentration of this metal reaches 0.1 mg/g of the body weight, it can be heavily lethal. Therefore, The United States Environmental Protection Agency (US-EPA) and World Health Organization (WHO) have set the maximum allowable concentration (MAC) for Cr at 0.1 and 0.05 ppm in drinking water, respectively [3,4].

The different methods namely, membrane filtration, electrochemical precipitation, ion exchange, adsorption, reduction of Cr(VI) to Cr(III), reverse osmosis, evaporation, chelating, solvent extraction, electrolysis and cyanide treatment were all employed for the removal of Cr(VI) from water and wastewater [1,2,5]. Most of these methods have some drawbacks such as low efficiency, high demand for energy, high cost, requiring special chemicals, and the problems related to the disposal of sludge [5,6]. While; the adsorption process due to its ease of operation, flexibility in design, low cost and high efficiency, has been effectively applied to removal of heavy metals including Cr(VI) [2].

In previous studies, various adsorbents such as granular activated carbon (GAC), powder activated carbon (PAC), mineral cartridge, biological and agricultural waste have been used for the removal of Cr (VI) [7–10]. Amongst these adsorbents, PAC due to its high porosity, large surface area and high efficiency has gained more interests than the others. In a comparative study by Jung et al., they compared the removal of Cr(VI) using PAC, chitosan, and single/multi-wall carbon nanotubes and found out that the maximum adsorption capacity of PAC (46.9 mg/g) was the highest within the studied adsorbents [5].

However, the main problem concerning PAC lies within its reusability and separation of it from aqueous solution. Thus, establishing the optimal conditions to facilitate the separation of PAC from the solution after the adsorption process seems to be essential. A way to achieve this purpose is to induce the magnetic properties into an adsorbent followed by the use of a magnet for physical separation. This so-called method has been widely used for the last few years due to its simplicity and high-speed [11,12]. Lv et al. [2] used nano Zero Valent Iron (nZVI)-Fe_3_O_4_ nanocomposite as an adsorbent for the removal of Cr(VI) and they demonstrated that 96.4% of Cr(VI) could be removed within 2 h under the conditions of pH 8.0 and initial Cr(IV) concentration of 20 mg/L. They also reported that the experimental data were fitted best to the pseudo second-order kinetic and Langmuir and Freundlich isotherm models [2].

Having used nZVI as iron source to magnetize PAC, we also used it as a reducing agent (E0 = −0.42 V) facilitating the reduction of Cr(VI) to Cr(III) according to the following reactions:

1 $$ {\mathrm{Fe}}^{\mathrm{o}} + {{\mathrm{H}\mathrm{CrO}}^{-}}_4+7{\mathrm{H}}^{+}\to\ {\mathrm{Fe}}^{3+} + {\mathrm{Cr}}^{3+} + 4{\mathrm{H}}_2\mathrm{O} $$

2 $$ {\mathrm{Fe}}^{\mathrm{o}} + {{\mathrm{Cr}\mathrm{O}}^{2-}}_4+8{\mathrm{H}}^{+}\to\ {\mathrm{Fe}}^{3+} + {\mathrm{Cr}}^{3+} + 4{\mathrm{H}}_2\mathrm{O} $$

3 $$ 2{\mathrm{Fe}}^{\mathrm{o}} + {\mathrm{Cr}}_2{{\mathrm{O}}^{2-}}_7+14{\mathrm{H}}^{+}\to\ 2{\mathrm{Fe}}^{3+} + 2{\mathrm{Cr}}^{3+} + 7{\mathrm{H}}_2\mathrm{O} $$

**Figure 1:**
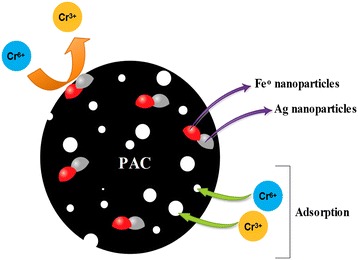
**Schematic of Cr(VI) adsorption and removal by PAC-Fe** ^**o**^ **/Ag composite.**

As shown in Figure 1, Fe^o^ has high affinity toward losing electron, for this reason, upon entering water it reacts with Cr(VI) and converts it to Cr(III). Then, Cr(III) is adsorbed by PAC and removed from the solution. Since the reactivity of nZVI is low, it is necessary to employ high active metals such as Ag, Pb, Ni and Cu in order to increase its catalytic capability [13]. These metals can accelerate the detoxification of Cr (VI) through protecting nZVI particles from the surface oxidation [3].

Herein, we used silver nanoparticles, due to their high electrochemical potential (E0 = 0.8), to enhance the catalytic ability of nZVI [14]. Having high specific area -at nanometer scale-, they could be used as a unique adsorbent for removal of pollutants [15,16]. Based on the above-mentioned findings, some researchers loaded Ag nanopartices on the adsorbents such as activated carbon and multiwall carbon nanotubes for the removal of dyes and heavy metals from the aqueous solutions [17,18].

So far, the removal of Cr(VI) using PAC-Fe^o^/Ag as an adsorbent has not been reported in the literature. This prompted us to combine the advantages of activated carbon and Fe^o^/Ag bimetallic nanoparticles for the preparation of magnetic composite PAC-Fe^o^/Ag as a new adsorbent for the removal of Cr(VI) from aqueous solutions.

## Material and method

### Adsorbent

All Chemicals used in this work were of analytical grade (>95% purity) and provided by Merck (Merck, Darmstadt, Germany). FeSo_4_.7H_2_O, NaBH_4_, HCl, NaOH and AgNO_3_ were used for the synthesis of PAC. Herein, we employed co-precipitation and reduction methods for the synthesis of PAC-Fe^o^ [19]. Initially, 10 g FeSO_4_·7H_2_O was dissolved in 200 mL of methanol:water (30:70, % v/v). Then, 10 g PAC was added into the resulting mixture. The pH of the mixture was adjusted at 7 with 3.8 M NaOH followed by the addition of an aqueous NaBH_4_ solution (4% w/v). After that, the whole mixture was stirred in a jar test for 45 min. The synthesized PAC-Fe^o^ particles were then separated from the liquid phase using a magnet (1.3 T) and washed at least three times with acetone and dried for 4 h under the N_2_-purged environment. The ferrous iron was reduced to ZVI and coated on PAC according to the following reaction Eq (4):

4 $$ 4{\mathrm{Fe}}_3 + {{3\mathrm{BH}}_4}^{-} + 9{\mathrm{H}}_2\mathrm{O}\ \to\ 4{\mathrm{Fe}}^{\mathrm{o}} + 3{\mathrm{H}}_2{{\mathrm{BO}}_3}^{-} + 12{\mathrm{H}}^{+} + 6{\mathrm{H}}_2 $$

In the next step, 10 g PAC-Fe^o^ powder was added to a diluted solution of AgNo_3_ (0.01 M) and the mixture was mixed on a shaker-incubator (HACH-HQ-USA) for 40 min at 300 rpm at 200 ± 1°C (flash mixing and high temperature) [20]. Finally, NaBH_4_ 3 M was added to complete the following reaction:

5 $$ 2{\mathrm{AgNo}}_3 + 2{\mathrm{Fe}}^{\mathrm{o}}\to 2\mathrm{Ag} + \mathrm{Fe}\ {\left({\mathrm{No}}_3\right)}_2 $$

The resulting bimetallic nanoparticles were separated by a magnet and immediately washed many times with water and finally dried under N_2_-purged for 2 h.

### Adsorbate

A stock solution of Cr(VI) (1000 mg/L) was prepared by dissolving the required amount of potassium dichromate (K_2_Cr_2_O_7_) in water and further diluted to prepare the solutions in the concentration range of 4–100 mg/L. The residual concentration of Cr(VI) was measured using a UV–VIS spectrophotometer (7400CE CECIL) at 540 nm by diphenylcarbazine method.

### Characterization of the synthesized adsorbent

The micro image, surface morphology, size and distribution of Fe^o^/Ag were analyzed by scanning electron microscopy (SEM, edxS360, Mv2300). The crystalline structure of the bimetallic nanoparticles coated on PAC was investigated by X-ray diffraction (XRD, Quantachrome, NOVA2000) using Cu-kα radiation and λ = 1.54Å 40kVp and 30 mA. The dimension and shape of the adsorbent was determined by transmission electron microscopy (TEM, PHILIPS, EM 208 S) with 100 keV.

### Batch adsorption experiments

All experiments were carried out under a batch condition using 100 ml Erlenmeyer flasks, each containing 50 ml of 4 mg/L Cr(VI) and a certain amounts of the adsorbent. The effect of pH in the range of 3–9 on the adsorption efficiency was studied under the following condition: contact time of 120 min and the stirring rate of 200 rpm. Herein, the pH of solution was adjusted using 0.1 M HCl or/and 0.1 M NaOH. The optimal contact time was then established under the condition of 0.3 g/L adsorbent, 4 mg/L Cr(VI) and room temperature. The Erlenmeyers were stirred in the range of 50, 100, 200, 300 and 400 rpm to determine the optimal agitation speed.

The effects of adsorbent dosage and initial concentration of Cr(VI) were examined in the range of 0.1-2 g/L and 4–100 mg/L, respectively. The effect of solution temperature on Cr(VI) removal efficiency was investigated at 25, 30, 40 and 50°C. It is worth noting that a shaker-incubator was employed to stabilize the temperature. All experiments were done in triplicate and the mean values were taken as the final results. The amount of Cr(VI) adsorbed onto the adsorbent at each contact time (q_e_) was calculated using the equation (6):

6 $$ {\mathrm{q}}_{\mathrm{e}}=\left({\mathrm{C}}_{\mathrm{o}}-{\mathrm{C}}_{\mathrm{e}}\right)\left(\frac{\mathrm{V}}{\mathrm{m}}\right) $$

Where C_0_ and C_e_ are initial and equilibrium concentration of Cr(VI) (mg/L), respectively. V is the volume of the aqueous phase (L) and m is the mass of PAC-Fe^o^/Ag (g).

### Adsorption isotherm

Adsorption isotherm describes the equilibrium of the adsorption material at the surface of adsorbent (i.e., at the surface boundary). Adsorption isotherms were obtained from the data derived from the regression analysis. The Langmuir and Freundlich isotherm models were used to evaluate Cr(VI) adsorption on the PAC-Fe^o^/Ag. The linear equations and parameters regarding the Langmuir and Freundlich isotherm models are presented in the Table 1.

**Table 1:** **The linear equations and parameters regarding Cr(VI) adsorption onto PAC-Fe** ^**o**^ **/Ag**

**Model**		**Linear equation**	**Parameters**
Isotherms	Langmuir	$\frac{{\mathrm{C}}_{\mathrm{e}}}{{\mathrm{q}}_{\mathrm{e}}}=\frac{1}{{\mathrm{q}}_{\mathrm{m}}{\mathrm{K}}_{\mathrm{L}}}+\frac{1}{{\mathrm{q}}_{\mathrm{m}}}{\mathrm{C}}_{\mathrm{e}}$	K_L_ and q_m_
	Freundlich	$\ln\ {\mathrm{q}}_{\mathrm{e}}= \ln\ {\mathrm{k}}_{\mathrm{f}}+\frac{1}{\mathrm{n}} \ln\ {\mathrm{C}}_{\mathrm{e}}$	K_F_ and n
Kinetics	Pseudo first-order	ln(q_e_ − q_t_) = ln q_e_ − k_1_t	q_e_ and k_1_
	Pseudo second-order	$\frac{\mathrm{t}}{{\mathrm{q}}_{\mathrm{t}}}=\frac{1}{{\mathrm{k}}_2{{\mathrm{q}}_{\mathrm{e}}}^2}+\frac{1}{{\mathrm{q}}_{\mathrm{e}}}\mathrm{t}$	q_e_ and K_2_

k_L_ (L/mg) is the empirical constant related to energy and q_m_ (mg/g) represents the maximum adsorption capacity. k_F_ and n are the Freundlich constants related to the adsorption capacity and intensity, respectively. The q_m_ and k_L_ parameters are calculated from the slope and intercept of the C_e_/q_e_ plot versus C_e_, respectively. The Freundlich isotherm parameters (k_F_ and n) are also calculated from the slope and intercept of the lnC_e_ plot versus lnq_e_, respectively.

The favorability of Cr(VI) adsorption onto PAC-Fe^o^/Ag was investigated using a dimensionless parameter, R_L_, derived from the Langmuir model. It expresses the essential characteristics of the isotherm model. R_L_ is defined as follows:

7 $$ {\mathrm{R}}_{\mathrm{L}}=\frac{1}{1+{\mathrm{k}}_{\mathrm{L}}{\mathrm{C}}_0} $$

Where, C_o_ is the initial concentration of Cr(VI). The adsorption will be favorable if R_L_ lies within 0 and 1. For R_L_ > 1, the adsorption is unfavorable; for R_L_ = 1 and 0, the adsorption is linear and irreversible, respectively [21].

### Kinetics of adsorption

Chemical kinetics deals with the experimental conditions influencing the rate of a chemical reaction. Herein, two kinetic models including the pseudo first-order and pseudo second-order models were applied for the modeling of the adsorption process of Cr(VI) onto PAC-Fe^o^/Ag. The linear equations of the mentioned models along with respective parameters are given in Table 1.

K_1_ (1/min) and k_2_ (g/(mg.min)) are the constant rate of the pseudo first-order and pseudo second-order models, respectively. The parameters related to the mentioned kinetic models can be obtained from the plots of ln(q_e_-q_t_) and q_t_/t against t.

### Thermodynamics of adsorption

Thermodynamics of adsorption is the systematic study dealing with the transformation of matter and energy in systems as they approach equilibrium state [12]. In thermodynamic studies, the determination of standard enthalpy (∆H^o^), standard free energy (∆G^o^) and standard entropy (∆S^o^) is necessary. The values of ∆H^o^, ∆S^o^ and ∆G^o^ are obtained by using the following equations:

8 $$ \ln {\mathrm{k}}_{\mathrm{d}}=\frac{\Delta {\mathrm{S}}^{\circ }}{\mathrm{R}}-\frac{\Delta {\mathrm{H}}^{\circ }}{\mathrm{R}}\frac{1}{\mathrm{T}} $$

9 $$ \Delta {\mathrm{G}}^{\circ }=\Delta {\mathrm{H}}^{\circ }-\mathrm{T}\Delta {\mathrm{S}}^{\circ } $$

10 $$ {\mathrm{K}}_{\mathrm{d}}=\frac{{\mathrm{q}}_{\mathrm{e}}}{{\mathrm{C}}_{\mathrm{e}}} $$

Where q_e_ is the amount of Cr(VI) adsorbed at equilibrium (mg/g) and C_e_ is the equilibrium concentration of Cr(VI) in solution (mg/L). R (8.314 J/mol K) is the universal gas constant and T (°K) is the solution temperature. The parameters of ∆H^o^ and ∆S^o^ can be obtained from the intercept and slope of the van’t Hoff plot (lnk_d_ versus 1/T), respectively.

## Results and discussion

### Adsorbent features

**Figure 2:**
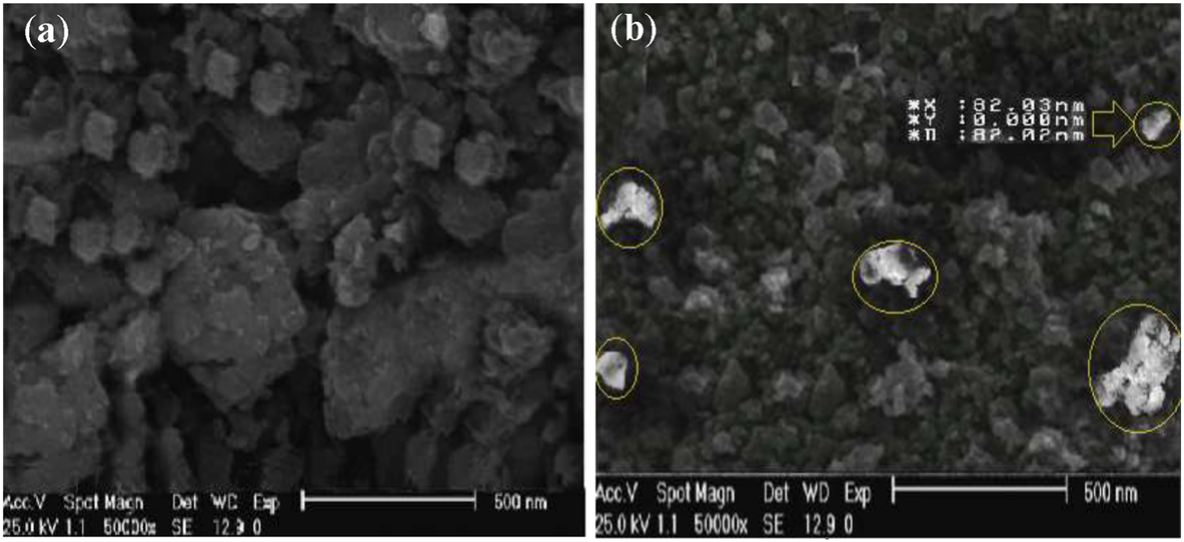
**SEM images of PAC (a) and PAC-Fe** ^**o**^ **/Ag (b).**

The morphology, size and surface of PAC and PAC-Fe^o^/Ag were analyzed by SEM. Figure 2(a) shows the image of SEM for PAC before being coated with Fe^o^/Ag bimetallic nanoparticles. It shows that the PAC has good porosity and high adsorption capacity. Figure 2(b) indicates the SEM analysis for PAC-Fe^o^/Ag, from which it can be deducted that the PAC structure is uniform compared with that of Figure 2(a). In Figure 2(b), white dots on the surface of the adsorbent represent the Fe^o^/Ag bimetallic particles which have an agglomeration structure and scattered abnormality. Figure 2(b) also shows that Fe^o^ and Ag particles were synthesized in nano scale (diameter of 82 nm).

**Figure 3:**
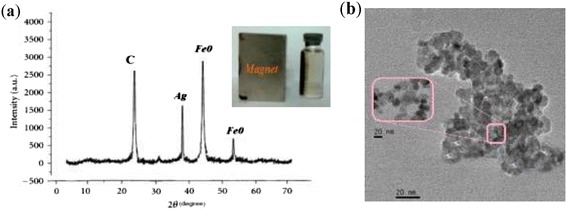
**XRD analysis of PAC-Fe** ^**o**^ **/Ag (a), magnetic separation of PAC-Fe** ^**o**^ **/Ag from aqueous solution (insert) and TEM image for PAC-Fe** ^**o**^ **/Ag (b).**

The XRD pattern of the synthesized adsorbent in the angle range of 2θ = 5-70°, applying Cu kα radiation (λ = 1.5A^o^) is shown in Figure 3(a). In this pattern, carbon (C) and silver (Ag) have been indicated at the peaks of 24.7° and 37.8°, respectively. Moreover, the peaks at the angles of 45.5° and 55.6° confirm the presence of Fe^o^ particles in the adsorbent structure. Generally, the XRD analysis confirmed that the Fe^o^ and Ag particles have been successfully coated on the PAC surface. Figure 3(a) also shows the magnetic properties of PAC-Fe^o^/Ag in magnetic separation process by using a magnet field.

The shape of Fe^o^/Ag bimetallic nanoparticles was analyzed by using TEM micrographs with 100 keV (Figure 3(b)). It can be deducted that the synthesized absorbent structure was polygon with irregular shape. Figure 3(a, insert) reflects the synthesized composite has a high magnetic sensitivity in the presence of an external magnetic field. Finally, it can be concluded that PAC-Fe^o^/Ag can be potentially applied as a magnetic adsorbent the for removal of Cr(VI) contaminants from aqueous solutions and, subsequently, the secondary pollution could be avoided.

### Effect of solution pH

**Figure 4:**
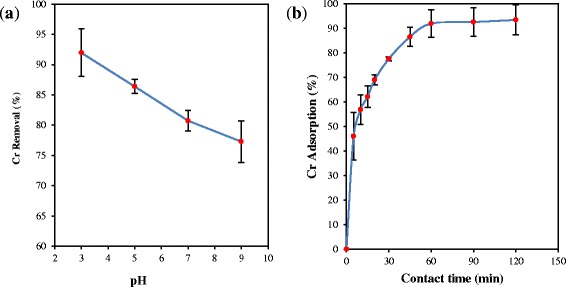
**Effect of pH (a) and contact time (b) on adsorption Cr(VI) onto PAC-Fe** ^**o**^ **/Ag (200 rpm agitation speed, 0.3 g/l adsorbent, 4 mg/L initial Cr(VI) concentration and 20 ± 1°C).**

The solution pH is one the main effective parameters which could have a significant role in controlling the adsorption process [22]. The effect of pH on Cr (VI) adsorption is shown in Figure 4(a). As shown in Figure 4(a), the maximum Cr(VI) removal occurred at acidic pH which can be due to the electrostatic attraction between the Cr(VI) anions and the positive charges located on the adsorbent surface. At acidic pH conditions, the predominant species of Cr(VI) come in various forms (Cr_2_O_7_^2−^, HCrO_4_^−^, Cr_3_O_10_^2−^ and Cr_4_O_13_^2−^), which they all bare negative charge(s) [3]. But, the fall in Cr(VI) removal as a result of the rise in pH may be due to the fact that at higher pH, the PAC-Fe^o^/Ag surface is negatively charged and subsequently enhances the electrostatic repulsion between Cr(VI) ions and the adsorbent, leading to the release of the adsorbed Cr(VI) species off the PAC-Fe^o^/Ag surface [1,23].

In addition; Since Fe^o^ particles could be easily oxidized to Fe^2+^ by Cr(VI) at pH < 6, they can promote the adsorption of Cr(VI). Therefore, it is concluded that the reduction process (i.e., the reduction of Cr(VI) to Cr(III)) at acidic condition promotes the efficiency of Cr(VI) removal, which was also suggested by other reports in the literature [3,4].

Since the maximum Cr(VI) adsorption (91.95%) was obtained at pH 3, this pH was selected as the optimum. This result is in good agreement with the previous studies [24,25]. In a further related studies, pH 3 was also reported as the optimal pH for the removal of Cr(VI) once nZVI–Fe_3_O_4_ nanocomposites, active carbon and saw dust adsorbents were employed [2,25,26].

### Effect of contact time

Figure 4(b) illustrates the effect of contact time on the Cr(VI) adsorption at the following condition: 0.3 g/l solution of the adsorbent, optimal pH (pH = 3.0 ± 0.1) and the contact time of 120 min. As indicated in Figure 4(b), the Cr(VI) adsorption efficiency was increased sharply up to 60 min and then it reached the equilibrium state right after 60 min. The sharp increase in the adsorption efficiency may be due to the existence of enormous vacant active sites in the adsorbent surface. However, by raising the contact time the availability of Cr(VI) ions to the active sites on the adsorbent surface is limited, which makes the adsorption efficiency reduce [21]. In a similar study, this phenomenon was investigated using different adsorbents [27,28]. In a further related study, Tang et al. reported that the adsorption of Cr(VI) on nano-carbonate hydroxyl apatite reached the equilibrium state at 90 min at different concentrations of Cr(VI) [29]. Since 90 min is more than the optimal time obtained in the present study, it can be noted that the PAC-Fe^o^/Ag has higher adsorption rate than nano-carbonate hydroxyl apatite.

### Effect of agitation speed

**Figure 5:**
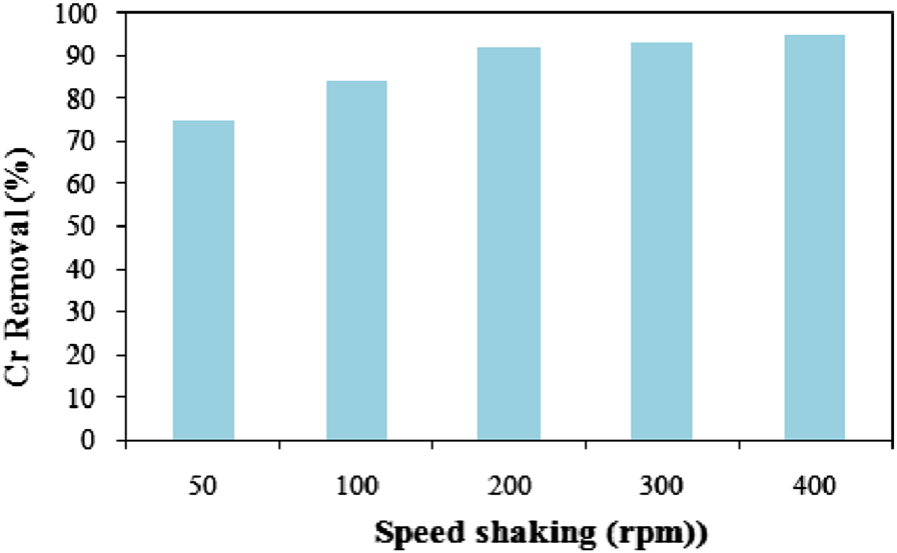
**Effect of agitation speed on Cr(VI) removal using PAC-Fe** ^**o**^ **/Ag (C**_**0**_ **= 4 mg/L, pH = 3.0 ± 0.1, contact time = 60 min, adsorbent dose = 0.3 g/L and 20 ± 1°C).**

In batch adsorption systems, agitation speed plays a significant role affecting the external boundary film and the distribution of the solute in the bulk solution [30]. The effect of agitation speed on Cr(VI) removal efficiency was examined in the range of 50–400 rpm (Figure 5). The results revealed that the Cr(VI) removal efficiency didn’t change beyond the agitation speed of 200 rpm. In a related study, Weng et al., reported that this phenomenon can be attributed to the little resistance of the boundary layer and high mobility of the system [30]. Hence, in the next experiments, the agitation speed of 200 rpm was selected as the optimal mixing speed.

### Effect of adsorbent dosage

**Figure 6:**
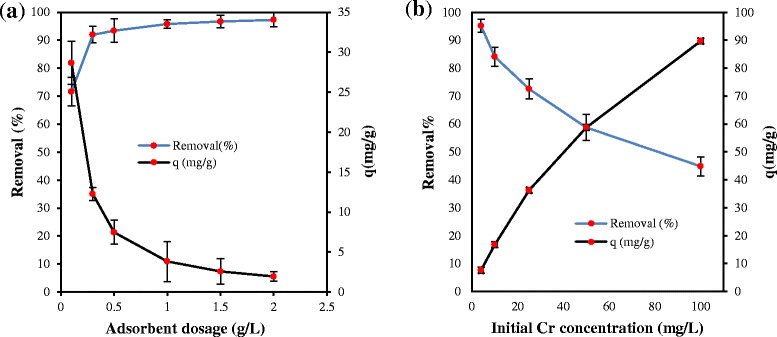
**Effect of adsorbent dosage (a) and initial Cr(VI) concentration (b) on removal efficiency and adsorption capacity of Cr using PAC-Fe** ^**o**^ **/Ag (pH =3.0 ± 0.1, contact time = 60 min and 20 ± 1°C).**

The effect of different amounts of PAC-Fe^o^/Ag on the adsorption capacity and efficiency under the optimal condition (pH = 3, t = 60 min and 200 rpm) is illustrated in Figure 6(a). It can be observed that with an increase in the adsorbent dosage from 0.1 to 2 g/l the removal efficiency increased from 71.60 to 97.25% for 4 mg/L of Cr(VI), while the adsorption capacity decreased from 28.64 to 1.95 mg/g. The rise in the adsorption efficiency is related to the increase in the availability of active sites on the PAC-Fe^o^/Ag, which can give rise to the adsorption of Cr(VI) ions [12].

Jung et al. [5] reported that with an increase in the dosage of various adsorbents, the Cr(VI) removal was enhanced [5]. However, a decrease in the adsorption capacity with an increase in the adsorbent dosage is probably due to instauration of the active sites on the adsorbent surface during the adsorption process. This phenomenon can also be due to the aggregation resulting from high adsorbate concentrations, leading to the decrease in the active surface area of the adsorbent [21].

### Effect of Cr different concentrations

Figure 6(b) shows the effect of different concentrations of Cr(VI) (4, 10, 25, 50 and 100 mg/L) on the efficiency of adsorption process. By increasing the initial Cr(VI) concentration from 4 to 100 mg/L, the percentage of adsorption decreased from 95.17 to 44.85%. The limit of active sites on the surface of adsorbent seems to be the main reason for the above-mentioned result [2,5]. Figure 6(b) also indicates that increasing the initial concentration of Cr(VI) has a positive impact on the adsorption capacity. This phenomenon may be attributed to the rise in the concentration gradient, which is similar to the findings by Cho and Luo [1,22].

### Adsorption isotherm

**Figure 7:**
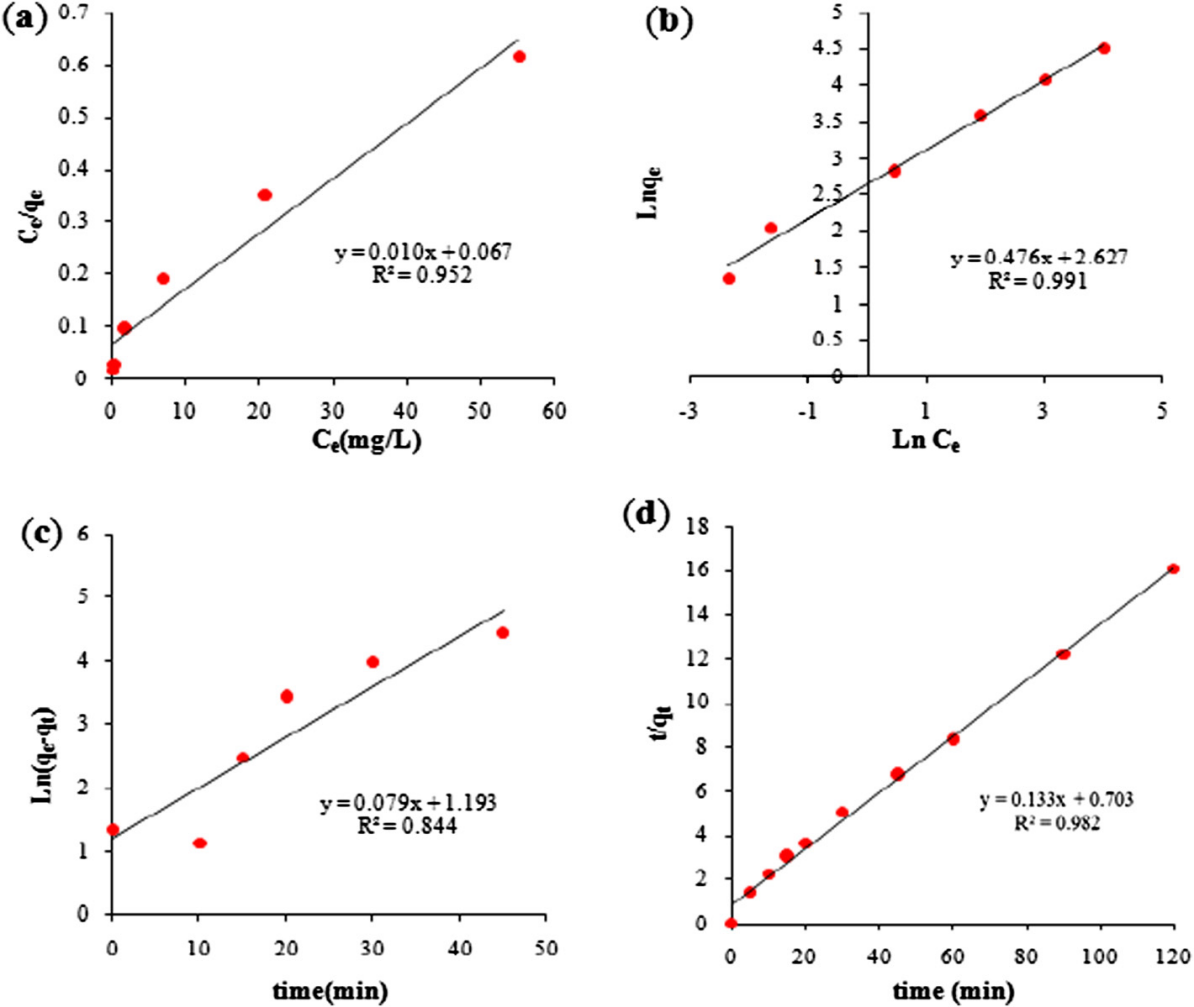
**The Langmuir (a), Freundlich (b) isotherm models and pseudo first-order (c) and pseudo second-order (d) kinetic models for the adsorption of Cr(VI) on PAC-Fe** ^**o**^ **/Ag.**

The obtained values regarding the Langmuir and Freundlich isotherms for Cr(VI) adsorption on PAC-Fe^o^/Ag at 25 ± 1°C are shown in Table 2. It is clear that the correlation coefficient (R^2^) for the Freundlich isotherm model (R^2^ > 0.99) is greater than that of the Langmuir isotherm model. This result reveals that the Freundlich model is in good agreement with the experimental data. The plots shown in Figure 7(a, b) also imply that the Freundlich model can be fitted to the experimental data. In fact, this model suggests that the active sites on the adsorbent surface are distributed in homogeneous form, and the adsorption of Cr(VI) on PAC-Fe^o^/Ag takes palace in a multilayer adsorption manner [31]. From Table 2, it is also observed that the values of R_L_ lie between 0 and 1, indicating that the Cr(VI) ions have been desirably adsorbed on PAC-Fe^o^/Ag [12]. Similar results have been reported by other researchers in the study of Cr(VI) adsorption on multiwall carbon nanotubes and the activated carbon produced from waste rubber tires [5,32].

**Table 2:** **The parameters regarding the adsorption isotherm models for Cr(VI) adsorption on PAC-Fe** ^**o**^ **/Ag**

**Isotherm models**	**Parameters**	
Freundlich	q_m_(mg/g)	100
	k_L_(L/mg)	0.15
	R^2^	0.952
	R_L_	0.625
Langmuir	k_f_(mg/g(Lmg)/n)	13.83
	n	2.1
	R^2^	0.991

Table 3 presents a comparison between the adsorption capacities of various adsorbents for the removal of Cr(VI). The maximum uptake of Cr(VI) per mass unit of PAC-Fe^o^/Ag was found to be 100 mg/g based on the Langmuir model. Also from Table 3, it is deducted that the activated carbon modified by nZVI and silver bimetallic nanoparticles has a good adsorption capacity compared to the other adsorbents.

**Table 3:** **Maximum adsorption capacities (q**_**m**_ **) of Cr(VI) on PAC-Fe** ^**o**^ **/Ag and the other adsorbents documented in the literature**

**Adsorbent**	**q**_**m**_ **(mg/g)**	**References**
PAC-Fe^o^/Ag	100.0	This study
Graphene oxide	65.2	[33]
Single-wall carbon nanotubes	20.3	[5]
nZVI–Fe_3_O_4_ nanocomposites	100.0	[2]
Activated carbon	3.46	[26]
Saw dust	20.70	[25]
Chitosan	35.6	[5]
MWCNTs (HNO_3_)	9.5	[34]
MnO_2_/Fe_3_O_4_/o-MWCNTs	186.9	[1]
Powdered activated carbon	46.9	[5]
Maghemite nanoparticles	19.2	[35]
Multi-wall carbon nanotubes	2.48	[5]

### Kinetics of adsorption

The kinetic models’ constant values of the adsorption process of Cr(VI) on PAC-Fe^o^/Ag along with their corresponding regression coefficients are given in Table 4. Based on the regression coefficient (R^2^), the adsorption kinetics of Cr(VI) can be better described by the pseudo second-order model. This result is also confirmed by the curves presented in Figure 7(c, d).

**Table 4:** **The parameters regarding the adsorption kinetic models of Cr(VI) on PAC-Fe** ^**o**^ **/Ag**

**Kinetic models**	**Parameters**		**q**_**e, exp**_
Pseudo first-order	q_e,cal_(mg/g)	3.3	7.22
	k_1_(min^−1^)	0.79	
	R^2^	0.844	
Pseudo second-order	q_e,cal_(mg/g)	7.51	
	k_2_(g/mg)(min^−1^)	0.025	
	R^2^	0.982	

The analysis of data from the pseudo second-order equation suggests that the adsorption of Cr(VI) onto PAC-Fe^o^/Ag is controlled by chemisorptions [21,36]. In addition, Table 4 also indicates that the adsorption capacity (q_e,cal_) calculated from the pseudo second-order model is well suited to the experimental data (q_e,exp_). Therefore, it can be concluded that the kinetics of Cr(VI) adsorption on PAC-Fe^o^/Ag fits best to the pseudo second-order model, which is in agreement with the previous reports on Cr(VI) adsorption [1,5,37]. This result also confirms that adsorption rather than reduction is more likely to be the predominant mechanism (i.e., the rate-limiting step of the process) [2].

### Thermodynamics of adsorption

**Figure 8:**
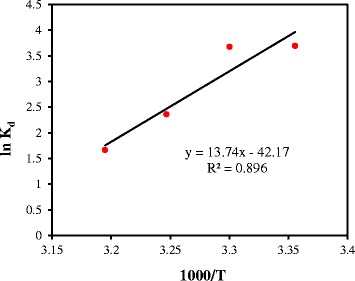
**Van’t Hoff curve for Cr(VI) adsorption on PAC-Fe** ^**o**^ **/Ag.**

The thermodynamic curves of Cr(VI) adsorption and the respective parameters are illustrated in Figure 8 and Table 5, respectively. It is noted in Table 5 that the amount of ∆H^o^ was found to be 146.99 KJ/mol. The positive value of ∆H^o^ indicates that Cr(VI) adsorption on PAC- Fe^o^/Ag is of an endothermic nature [18]. On the other hand, the negative value of ∆G^o^ indicates that the Cr(VI) adsorption process is spontaneous [1,2]. According to Table 5, there is an inverse relationship between the temperature and the amount of ∆G^o^, which reveals that the adsorbent shows better performance at higher temperatures [18,21]. The amount of ∆S^o^ was also found to be negative (−0.451 KJ/mol), indicating that with increasing the temperature the adsorption efficiency decreases in solid/liquid phases [12].

**Table 5:** **The values of thermodynamic parameters of Cr(VI) adsorption on PAC-Fe** ^**o**^ **/Ag**

**Temperature(°K)**	**lnk**_**d**_	**∆G** ^**o**^ **(kJ/mol)**	**∆H** ^**o**^ **(kJ/mol)**	**∆S** ^**o**^ **(kJ/mol.K)**
298	3.69	−9.25	146.9	−0.45
303	3.67	−9.14		
313	2.36	−6.04		
323	1.66	−4.33		

## Conclusions

In the present study, the synthesized bimetallic nano composite (PAC-Fe^o^/Ag) was used as an adsorbent for the removal of Cr(VI) from the aqueous solutions. The results illustrated that the synthesized adsorbent showed a high efficiency in adsorption of Cr(VI). The optimum conditions for the adsorption process obtained at acidic pH (pH = 3), the contact time of 60 min and the temperature of 50°C. Moreover, the equilibrium and kinetic studies indicated that the Cr(VI) adsorption followed the Freundlich isotherm and pseudo second-order kinetic models. The values regarding the thermodynamic parameters also implied that the adsorption of Cr(VI) was spontaneous and endothermic in nature. Due to favorable performance of PAC-Fe^o^/Ag in the removal of Cr(VI) and its feasible separation from the aqueous solutions, it can be used as an efficient adsorbent in the treatment of water and wastewater with no need of further filtering and centrifugation, etc., and also it could be used as an alternative to activated carbon.
